# Green Synthesis of Mg_0.99_ Zn_0.01_O Nanoparticles for the Fabrication of *κ*-Carrageenan/NaCMC Hydrogel in order to Deliver Catechin

**DOI:** 10.3390/polym12040861

**Published:** 2020-04-09

**Authors:** Farzaneh Sabbagh, Khadijeh Kiarostami, Nadia Mahmoudi Khatir, Shahabaldin Rezania, Ida Idayu Muhamad

**Affiliations:** 1 Department of Botany and Plant Science, Faculty of Biological Science, Alzahra University, Tehran 1993891176, Iran; f.sabagh@alzahra.ac.ir; 2 Department of Biotechnology, Faculty of Biological Science, Alzahra University, Tehran 1993891176, Iran; n.khatir@alzahra.ac.ir; 3 Department of Environment and Energy, Sejong University, Seoul 05006, Korea; 4 Department of Bioprocess & Polymer Engineering, Faculty of Engineering, Universiti Teknologi Malaysia, Johor 81310, Malaysia; idaidayu@utm.my

**Keywords:** catechin, κ-Carrageenan, sol-gel, nanoparticle, green synthesis

## Abstract

Currently, the role of the nanoparticles in the structure of the composites and their benefits for the health of the body is valuable. In this study, the effects of the doping on the structural and morphological properties of the hydrogels using a Mg co-doped ZnO hydrogel, which has been fabricated by the sol–gel process, have been investigated. Then, a hydrogel containing nanoparticle and a hydrogel without any nanoparticles was produced as a control. The hydrogels were loaded with catechin and the related characterization was evolved based on the new structure of the matrices. The Mg_0.99_Zn_0.01_O nanoparticles were synthesized using a green synthesis method. To investigate the properties of the nanoparticles, zeta potential and XRD were studied. The field emission scanning electron microscopy (FESEM), FTIR, TGA, swelling Ratio, and compression tests were investigated for the hydrogels. Based on the results, FESEM showed a more compressed structure for hydrogels including nanoparticles rather than the hydrogels without a nanoparticle. The TGA showed a higher decomposition temperature in the hydrogels including nanoparticles. The swelling ratio of hydrogels containing a nanoparticle was higher than the control hydrogel. κ-Carrageenan/ Mg_0.99_Zn_0.01_O/NaCMC/Catechin had the highest swelling ratio (44.15%) rather than the κ-Carrageenan/NaCMC (33.22%). Mg_0.99_Zn_0.01_O nanoparticles presented a stronger structure of hydrogels in the compression test. It is concluded that the role of the synthesized nanoparticle is critical in the structure of the hydrogel.

## 1. Introduction

Hydrogels are polymer networks that keep a huge amount of water and present a semi-solid morphology. They have a three-dimensional network caused by physical or chemical crosslinking interactions [1]. Catechin is one of the most important ingredients of green tea [2]. Flavonoids are from the secondary metabolism of plants and are widely found in the realm of plants. They are divided into six groups of flavonons, flavans, isoflavones, flavanols, and anthocyanins based on their structure and position of the heterocyclic oxygen ring [3]. Catechins are antioxidants, which consisted of 25% to 35% of the dry weight of green tea and have beneficial effects on the human body. Catechin has the property of inhibiting free radicals and acts as a biological antioxidant [4]. Biological properties of tea like the antioxidant, antimicrobial, anti-cancer, and anti-mutagenic properties are related to catechins. They naturally have demonstrated potential in both treatment and prevention and a wide range of diseases including cardiovascular diseases, Parkinson’s disease, cancer, diabetes, osteoporosis, and Alzheimer’s disease [5]. In addition to the direct effects of antioxidants of catechins, they can indirectly increase the body’s androgenic antioxidants [6]. In a research study, mice who received the extracted green tea have shown the increased levels of intrinsic antioxidants such as glutathione peroxidase, reductase, superoxide dismutase, and catalase [7].

Nanoparticles are particles smaller than 100 nm in diameter [8]. Nanomaterials can easily cross cellular barriers compared to larger particles and move to a cell or organ due to their special properties [9]. Pure hydrogels have a low mechanical strength. To optimize the thermal, mechanical, optical, and chemical properties of hydrogels, it is necessary to use-nanoparticle-loaded hydrogels. Nanoparticle-loaded hydrogels present many benefits in drug delivery such as improvement of bioavailability and efficacy and safety of the drugs (catechin) by providing targeted delivery of drugs, and or extending drug in the target tissue. Based on the methodology of nanoparticles preparation, the drug (catechin) is dissolved, entrapped, or encapsulated into a nanoparticle matrix [10]. The unique size-dependent characteristics of metal nanoparticles such as MgO and ZnO have made them interesting. They are capable of modifying the nanostructures to more suitable materials for biological systems such as biocompatibility, surface layer modification for more solubility, and biorecognition [11].

One of the functional applications of nanoparticles in industry and medicine is magnesium oxide (MgO). The results of studies show that magnesium oxide nanoparticles act as a potentially effective antibacterial agent and are used alone or in combination with other antimicrobial groups [10]. On the other hand, in many fields such as medicine, ZnO nanoparticles have been used for its photocatalytic and antimicrobial properties. Zinc oxide nanoparticles can be used as photocatalytic pigments, chemical sensors, drug carriers in targeted drug delivery, and the provision of cosmetics such as sunscreens [11].

It is important to produce zinc oxide nanoparticles that utilize cost-effective methods to produce uniform nanoparticles [12,13]. On the other hand, one of the problems that threaten the environment and human health is the use of hazardous materials and reagents in preparing various materials including nanomaterials. Therefore, the synthetic methods based on the principles of green chemistry at the laboratory and industrial scale have been developed. The researchers synthesized zinc oxide nanoparticles using sol-gel techniques and natural materials. The most prominent features of this method are simple synthesis, usage of low-cost materials, compliance with the principles of green chemistry that lead to avoiding using hazardous materials, and references to human health and the environment [14].

NaCMC has a polar structure of carboxyl groups and is a soluble material in hot and cold water that can form clear mucilage. The important application of this material is to use in film-forming, gel production, wound dressing, and suspensions [15].

κ-Carrageenan is a linear, sulfated polysaccharide with repeating 3,6-anhydro-D-galactose and D-galactose units that exist in many kinds of seaweed and forms thermos reversible gels in the presence of cations in aqueous solutions. Additionally, they can be used to modify the rheological characteristics of solutions. This polymer is a good selection for gel production, stabilize the disperse systems, and modify the viscosity [16].

The current research study is aimed at producing a κ-Carrageenan/Mg_0.99_Zn_0.01_O /NaCMC hydrogel based on the green synthesis of nanoparticles. The nanoparticle and polymer were synthesized and characterized separately. Then, catechin-loaded polymers were prepared and their physicochemical characterization have been analyzed.

## 2. Materials and Methods

### 2.1. Chemicals

The κ-Carrageenan, calcium carbonate (CaCO_3_), Sodium carboxymethyl cellulose (NaCMC), (+)-Catechin hydrate, Zinc nitrate hexahydrate, Magnesium nitrate hexahydrate, and gelatin, which have been used in this study were procured from Sigma-Aldrich (Jefferson City, MS, USA). All these materials were applied as received without any additional purification. Distilled water was applied in the synthesis of the hydrogel.

### 2.2. Methods

#### 2.2.1. Particles Preparation and Characterization

**Scheme 1:**
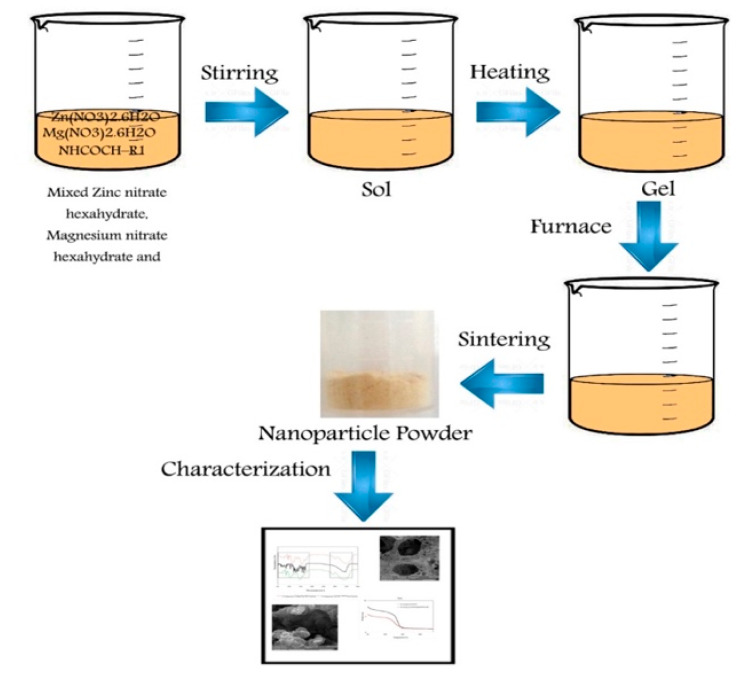
Synthesis of the nanoparticle with a sol-gel method.

To prepare Zn-doped MgO nanoparticles (NPs) in the form of Mg_1-x_Zn_x_O Magnesium, nitrate hexahydrate (Mg (NO_3_)_2_.6H_2_O), Analytical grade Zinc nitrate hexahydrate (Zn (NO_3_)_2_.6H_2_O), gelatin [(NHCOCH–R1) n, R_1_= amino acid, Type A, Porsin], and distilled water were used. To produce 2 g of the final composition of MgO nanoparticle, 12.725 g of (Mg (NO_3_)_2_.6H_2_O) was used. In order to produce Mg_0.99_Zn_0.01_O, 12.674 g (Mg (NO_3_)_2_.6H_2_O) and 0.148 g (Zn (NO_3_)_2_.6H_2_O) dissolved into 20 mL of distilled water. Alternatively, 4 g of gelatin (the ratio of gelatin to the final product was 2:1) was gradually added to 60 mL of distilled water and the solution was continuously stirred at 80 °C in an oil bath. Scheme 1 presents the process of nanoparticle production. Using the Mg_1-x_Zn_x_O formula, the number of nitrates was calculated, where x is equal to 0, and 0.01. A clear solution was attained after the dissolution of gelatin in the water. Then, the Mg^2+^ and Zn^2+^ solutions were added to the solution of gelatin. A transparent, viscous, and honey-like gel was obtained due to continuously stirring of the solution for 7 h at 80 °C. Lastly, for the calcination process, a small amount of provided gel was rubbed on the internal walls of an alumina crucible before being placed into the 600 °C furnace. The heating rate was 5 °C/min and the heating operation was for 2 h [17].

#### 2.2.2. Zeta Potential

By using a Zeta CAD (CAD, Les Essarts le Roi, France), the zeta potential analysis of the Mg_0.99_Zn_0.01_O nanoparticles were measured. The preparation of the nanoparticles was held at a pH of 7, and the samples were examined at least three times [18].

#### 2.2.3. X-ray Diffraction (XRD) Analysis

X-ray diffraction (XRD) analysis of samples was carried out by a powder diffractometer (SEIFERT PTS 3003, Rigaku, Ultima IV, Japan) using a Cu Kα radiation (λ = 1.54 A°) [19].

#### 2.2.4. Morphology of Nanoparticles

The morphology of nanoparticles was evaluated using field emission scanning electron microscopy (FESEM). For FESEM, gold-coated nanoparticles were mounted under vacuum, on a holder, and then assessed in a LEO 1450 VP SEM (20 kV, Ramsey, NJ, USA) [17].

#### 2.2.5. Hydrogel Preparation

To prepare the hydrogels, 0.48 g of κ-carrageenan and 0.12 g NaCMC was dissolved in 20 mL of distilled water at 80 °C before mixing with 0.15 g CaCO_3_, which was dissolved in 10 mL of distilled water. To improve the swelling content of the hydrogels, NaCMC was merged with κ-carrageenan. Stirring the solution for 1 h is needed to achieve a viscous, clear, and homogenous solution without any bubble [19]. Mg_0.99_Zn_0.01_O aqueous solution was prepared with 0.8 mg/mL concentration. As a result, the addition of Mg_0.99_Zn_0.01_O to κ-Carrageenan solution by stirring at room temperature formed the Mg_0.99_Zn_0.01_O / κ-Carrageenan [20].

#### 2.2.6. Loading of Catechin in the Hydrogel

By dissolving 150 mg/mL of catechin in 5 mL of distilled water and adding it to 25 mL of hot NaCMC/CaCO_3_ solution at 80 °C, the catechins were dissolved. This solution was stirred for 1 h to gain a transparent, smooth, thick, and bubble-free solution. Then, the hydrogel should be placed in this solution for 24 h at 4 °C [21].

#### 2.2.7. FTIR

FTIR spectroscopy was used to identify the functional groups of the samples. It was assessed using an FTIR (Thermo Nicolet Avatar 370, BioSurplus, Inc. San Diego, CA, USA). Potassium bromide was mixed with powdered samples and was used to prepare the tablets. The wavelength of 400–4000 cm^−1^ was used for the FTIR spectra [22].

#### 2.2.8. Thermo Gravimetric Analysis (TGA)

The thermogravimetric analysis of hydrogels was carried out using a Shimadzu apparatus TGA-50 (Shimadzu, Tokyo, Japan) by the weight of sample, which is 5–10 mg within 30–800 °C at the heating rate of 10 °C/min under nitrogen flow (20 mL/min) [23].

#### 2.2.9. Compression Test

The tests on the hydrogels were evaluated using a texture analyzer (TA. XT. Plus, Stable Micro Systems Ltd., Godalming, UK). The hardness, adhesion, and springiness of the hydrogels were measured. The analytical probe P/0.4 (stainless steel cylinder, 4 mm in diameter) was driven through the hydrogels (20 mm in height) to a depth of 60% of the sample height at a speed of 1.0 mm/s and with a trigger force of 3 g for three times [24].

#### 2.2.10. Swelling Ratio

About 2.0 g of samples were weighed and then immersed in distilled water at room temperature. The samples were removed from the water periodically, dried and were re-weighed respectively. The swelling of hydrogels was calculated using the following equation.

Swelling ratio%= (W_s_ − W_0_)/W_0_ × 100 (1)

where W_s_ and W_0_ are the weight of swollen hydrogel at time (t) and initial weight of hydrogels respectively [20].

#### 2.2.11. Statistical Analysis

At least three replications were performed for all the experiments. A completely randomized design was applied for the statistical analysis. All formulations were expressed as mean ± standard deviation. A one-way analysis of variance (ANOVA) with the “Tukey’s post hoc test” for multiple comparisons by using SPSS software (SPSS, version 18), was used to check a statistically significant difference between samples. A *p*-value less than 0.05 was considered as statistically significant.

## 3. Results

### 3.1. Zeta Potential

**Figure 1:**
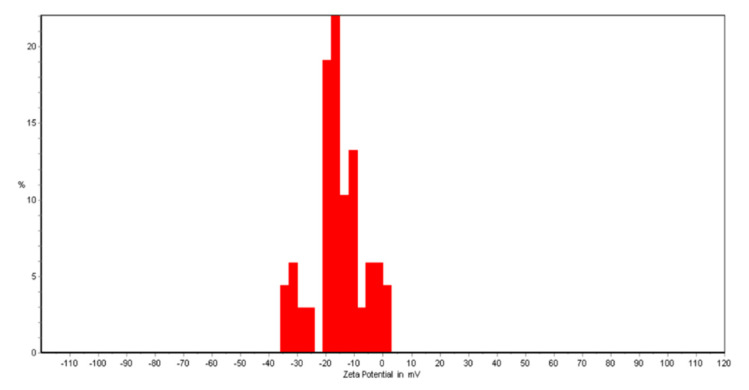
Zeta potential of Mg_0.99_Zn_0.01_O nanoparticle at a pH of 7.

The zeta potential characterizes electrochemical surface properties when functional groups dissociate on the surface or ions transfer to surfaces from the inner layers. Then, the electrostatic interaction of surface molecules of Mg_0.99_Zn_0.01_O and the zeta potential (ZP) analysis are shown in Figure 1. Generally, Wurtzite-type ZnO nanoparticles have positive surface charges in the as-prepared state [25]. However, one of the required key elements for oxygen evolution is the ability to use Mg ions as rapid secondary electron donors [26]. Functional ions transferring to the surface is affected by varying the Mg concentration value of the component. Increasing a net charge at the particle surface influences the ion distribution in the surrounding interfacial region, which increases the concentration of counter ions close to the surface.

### 3.2. XRD Patterns of Hydrogels and Nanoparticles

**Figure 2:**
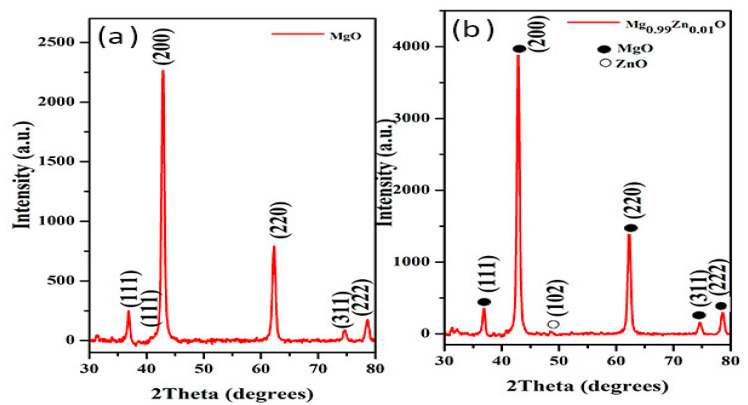
XRD pattern of (**a**) MgO and (**b**) Mg_0.99_Zn_0.01_O.

The MgO nanoparticle has stability and is suitable for human health as they are necessary minerals. On the other hand, the ZnO nanoparticle has superior antibacterial and antimicrobial properties [26,27,28,29]. The potential use of ZnO nanoparticles in biomedical applications is gaining interest in the scientific and medical communities due to their physical and chemical properties [30]. In this work, using Zn-doped MgO nanoparticles instead of simple ZnO or MgO nanoparticles was preferred. In Figure 2, the sharp and intense peaks indicated that the samples were highly crystalline as compared to simple ZnO or MgO nanoparticles.

**Figure 3:**
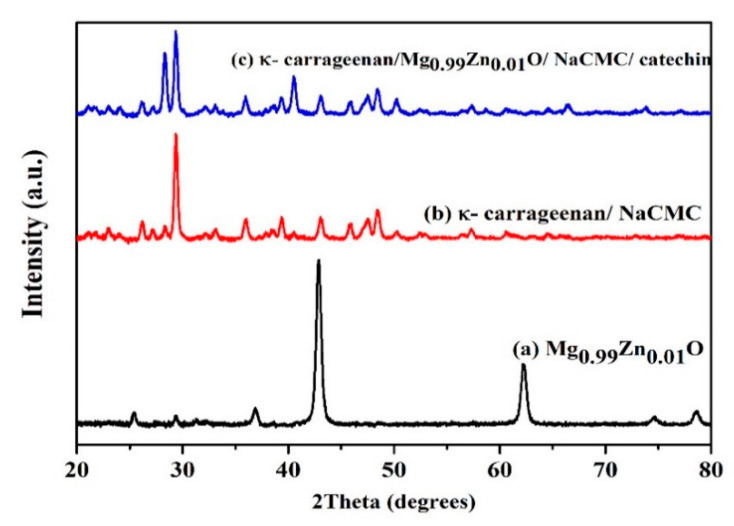
XRD patterns of (**a**) Mg_0.99_Zn_0.01_O, (**b**) κ-carrageenan/NaCMC, and (**c**) κ-carrageenan/Mg_0.99_Zn_0.01_O/NaCMC/Catechin.

Figure 3 presents the XRD patterns of the samples. The crystalline property of Mg_0.99_Zn_0.01_O nanoparticle (Figure 3a), κ-Carrageenan/NaCMC hydrogel (Figure 3b), and the κ-Carrageenan/ NaCMC/ Mg_0.99_Zn_0.01_O /Catechin hydrogel (Figure 3c) was performed by XRD analysis in Figure 3. The results showed that the MgO cubic structure and ZnO hexagonal structure were formed at the selected calcination temperature as 650 °C. Hence, some new diffraction peaks were observed in the patterns as catechin added to the compound. The peaks that appeared at 2θ = 29°, 35°, 43°, and 47° were indexed to the hexagonal crystalline structure. In other words, most ZnO atoms were defused in the cubic structures of ZnO nanoparticles and formed as ZnO nanostructures in addition to the MgO nanoparticles.

**Figure 4:**
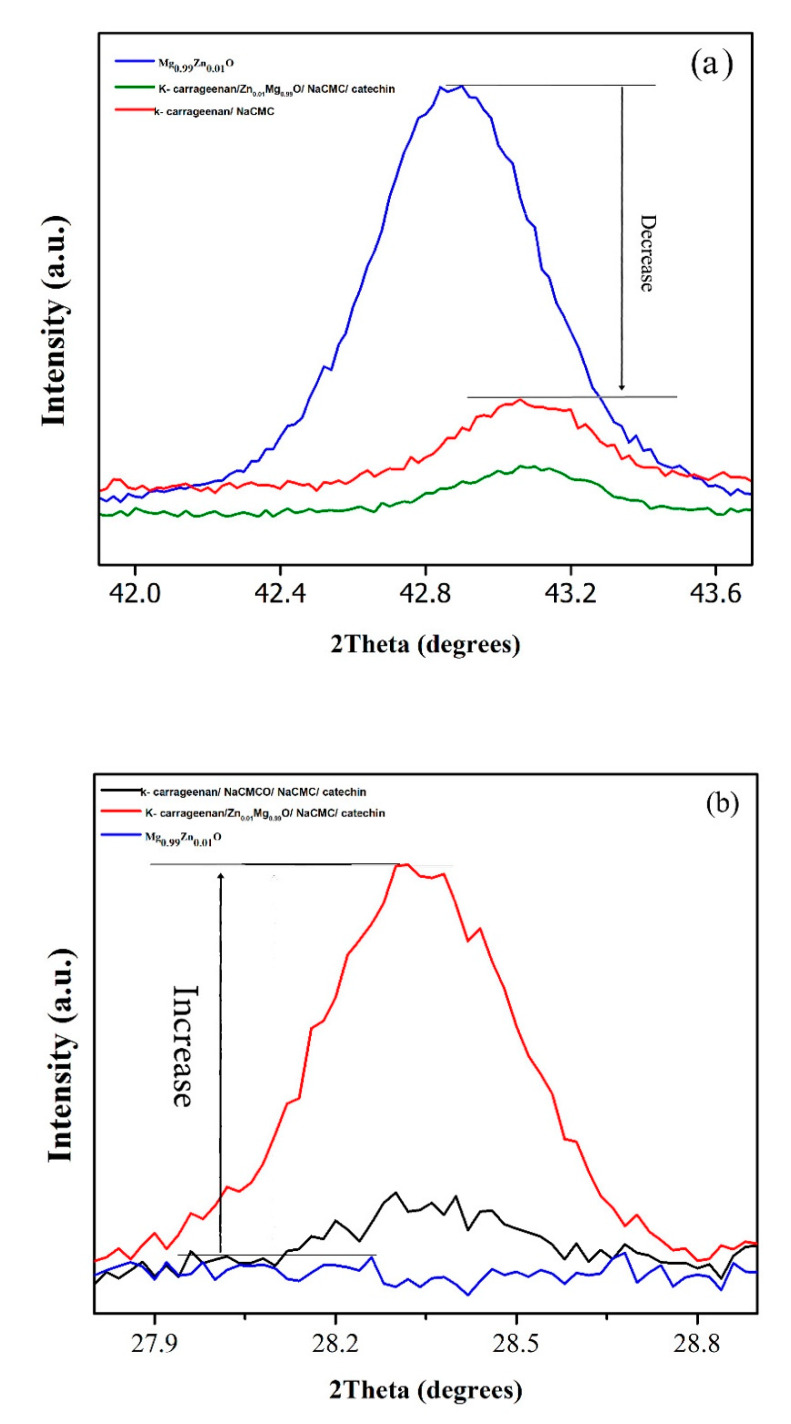
XRD peak related to (**a**) MgO cubic and (**b**) ZnO hexagonal structures.

Figure 4a,b show the diffraction peaks related to MgO-nanoparticles and Catechin κ-carrageenan/NaCMC, κ-carrageenan/NaCMC/Mg_0.99_Zn_0.01_O/Catechin. As expected, the intensity of the diffraction peak increased as the ZnO amount increased in the compound (Figure 4b). The estimated crystalline sizes for Mg_0.99_Zn_0.01_O and κ-carrageenan/NaCMC/Mg_0.99_Zn_0.01_O/Catechin were 18 and 20 nm, respectively.

**Figure 5:**
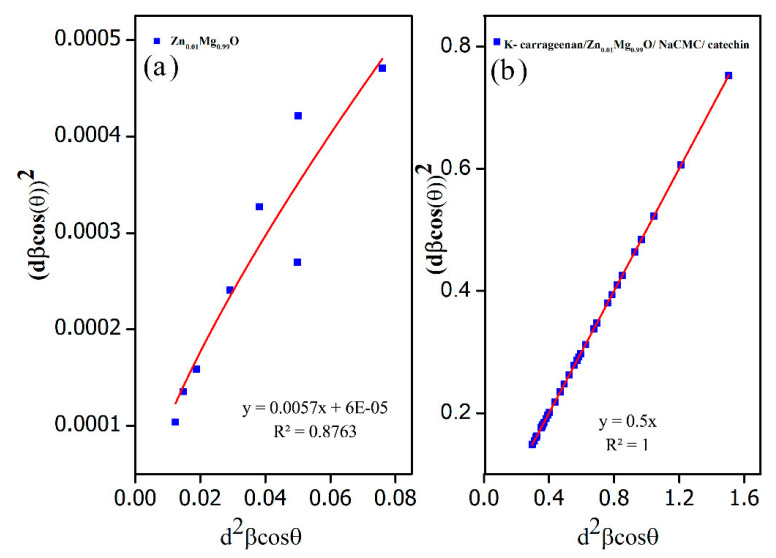
SSP plot for (**a**) Mg_0.99_Zn_0.01_O, (**b**) κ-carrageenan/NaCMC/Mg_0.99_Zn_0.01_O/Catechin.

Figure 5a,b are associated with the MgO nanoparticle cubic structure and hexagonal ZnO nanocrystals, respectively. As can be seen in Figure 5a, the intensity of the diffraction peaks was decreased as the Mg atoms were added to the compound due to the occurred defects in the cubic structure by adding impure Mg. The XRD patterns of κ-Carrageenan/NaCMC hydrogel showed many extensive diffraction peaks at 30°, which suggests that it had an amorphous property. The addition of Mg_0.99_Zn_0.01_O nanoparticles to the κ-Carrageenan/NaCMC hydrogel did not change the XRD pattern of the hydrogel remarkably, which indicates acceptable compatibility among κ-Carrageenan/NaCMC and the Mg_0.99_Zn_0.01_O nanoparticle. This result is in line with the findings of Zohourvahid-Karimi et al. [30]. They produced κ-Carrageenan/PFE (polyphenol-rich pomegranate flesh extract) and κ-Carrageenan/PPE (pomegranate peel extract) films. By the addition of PPE and PFE to κ-Carrageenan, the pattern of XRD had not remarkably changed [31].

Some methods can be used for calculating the crystalline size such as Scherrer and size-strain plot (SSP). In the Scherrer method, the considered sizes are 18 and 20 nm for κ -carrageenan/NaCMC/Mg_0.99_Zn_0.01_O/Catechin and Mg_0.99_Zn_0.01_ O, respectively. SSP methods are more common and powerful methods than the Scherrer method since the strain of the lattice affecting the intensity and position of the diffraction peaks is considered for the calculations. The obtained XRD data were analyzed by the SSP method. In this method, ${\left(d_{hkl}\beta_{hkl}cos\theta \right)}^{2}$ is plotted regarding $\left(d_{hkl}^{2}\beta_{hkl}cos\theta \right)$ as the following relation.

$${\left(d_{hkl}\beta_{hkl}cos\theta \right)}^{2}=\frac{A}{D}\left(d_{hkl}^{2}\beta_{hkl}cos\theta \right)+{\left(\frac{\varepsilon }{2}\right)}^{2}$$

where A is the plane distance, β is the full peak width at half minimum intensity (FWHM), θ is the peak position, D is the crystalline size, and ε is the lattice strain. A is a constant equal to ¾. The crystalline size was estimated from the slope of the fitted data.

### 3.3. FESEM

**Figure 6:**
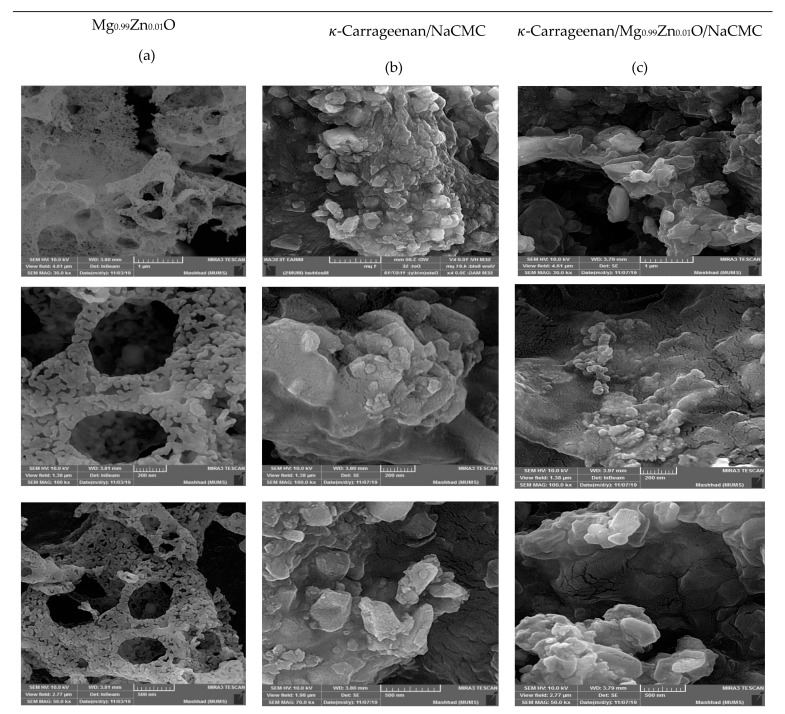
FESEM images of (**a**) Mg_0.99_Zn_0.01_O, (**b**) κ-carrageenan/NaCMC, and (**c**) κ-carrageenan/Mg_0.99_Zn_0.01_O/NaCMC.

To verify the effect of Mg_0.99_Zn_0.01_O nanoparticles on the microstructure of hydrogels, the surface morphological properties of the Mg_0.99_Zn_0.01_O nanoparticle and hydrogels were analyzed using FESEM. The FESEM images of the samples are shown in Figure 6. Generally, to produce nanoparticles by using the sol-gel method, some wrinkle networks appear on the surface including spherical nano-sized crystallites [32,33,34]. Although, the surface properties can be influenced by the incorporation of dopant. The type of dopant is an important factor on the surface properties. Based on the results, the incorporation of Mg_0.99_Zn_0.01_O ions improved the surface quality of the hydrogel by giving a regular grain size to produce a more uniform hydrogel. The addition of Mg_0.99_Zn_0.01_O changed the surface morphology of hydrogels [35]. The surface morphology of the control hydrogel (κ-Carrageenan/NaCMC) was changed by loading the Mg_0.99_Zn_0.01_O nanoparticles. The results showed that the addition of Mg_0.99_Zn_0.01_O (Figure 6a) to the hydrogel (Figure 6b) made the surface of the nanocomposite more compact (Figure 6c) rather than the hydrogel without Mg_0.99_Zn_0.01_O. Nanoparticles located inside the network of the hydrogel play a role as nano-sized reservoirs. The enormous interphase area between the network and nanoparticles leads to the reduction of the typical diffusion length of water molecules in the hydrogels. Such water-friendly reservoirs could dehydrate fast and play a role as channels for water released from the hydrogel network [36,37].

### 3.4. FTIR

**Figure 7:**
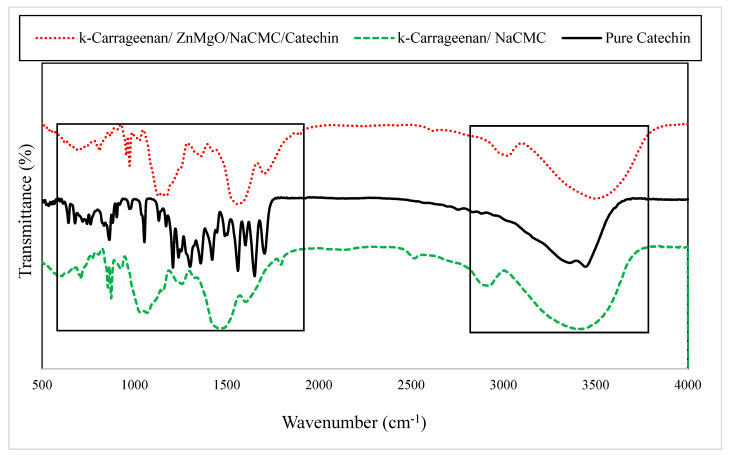
FTIR spectra of κ-Carrageenan/ Mg_0.99_Zn_0.01_O /NaCMC/Catechin, κ-Carrageenan/NaCMC, and pure (+)-catechin.

In Figure 7, the FTIR spectrum of the κ-Carrageenan/NaCMC hydrogel showed the peak at 3398 cm^−1^ due to the O-H stretching. The peak that appears at 2925 cm^−1^ corresponds to the C-H stretching vibrations of alkane groups. The peaks observed at 1259 cm^−1^ was due to the S-O of sulfate esters. Another peak observed at 923 cm^−1^ corresponds to the 3,6-anhydro-D-galactose. A peak observed at 856 cm^−1^ was due to the galactose-4-sulfate. Similarly, Tanusorn et al. [38] produced a P3HT/carrageenan hydrogel and the results of FTIR analysis of the carrageenan were close to this study. FTIR spectra of (+)-catechin sample are shown in Figure 7 for the commercial sample as received. There were many characteristic peaks of different intensity between 604 and 3406 wavenumbers (cm^−1^) [39] and an adsorption OH group peak at 3406 cm^−1^. It should be noted that interpretation of the 1665–604 cm^−1^ regions is difficult. This region is called the” fingerprint region” where many different vibrations take place. The exact value of the peak wavenumber and their intensities for (+)-catechin were as follows: the peaks observed at 702–1094 cm^−1^ were related to the benzene ring (1,2-distributed and 1,3-distributed). Another peak at 1261 cm^−1^ was related to the C-O alcohol ingredient. The C=O linkages are verified at 1665 cm^−1^. The peaks that appeared at 1400 cm^−1^ to 1600 cm^−1^ correspond to the C=C stretching vibration of the aromatic and alkane medium. The characteristic peaks at 2843–3313 cm^−1^ correspond for alkane medium and the peaks at 3313–3406 cm^−1^ correspond to aromatic mediums [40]. The spectra of Mg_0.99_Zn_0.01_O in combination with κ-Carrageenan and catechin is also presented in Figure 7. A strong peak at around 3398 cm^−1^ was related to the stretching vibration of the –OH bond of H_2_O in the Mg_0.99_Zn_0.01_O lattice due to the moisture in the atmosphere and solution [41]. The existence of H_2_O on the surface of Mg_0.99_Zn_0.01_O nanocrystals is due to such vibrations [42]. The absorption bands at 2918 cm^−1^ and 2924 cm^−1^ performed in the spectrums of the hydrogel are related to stretching frequency of –CH_3_ groups [43]. One peak at 1455 cm^−1^ is due to the stretching of zinc carboxylate [44].

### 3.5. Compression Test

To evaluate the textural characteristics of the hydrogels, the compression test was performed. Table 1 presents the result of Mg_0.99_Zn_0.01_O on the textural properties of the hydrogel. The hardness of the κ-Carrageenan/Mg_0.99_Zn_0.01_O/NaCMC hydrogel reached 77.85 ± 3.40 (g). However, before the addition of the nanoparticle to the complex, the hardness was 76.61 ± 2.34 (g). The springiness of the hydrogels in both compositions was constant and no specific difference was observed. The addition of the nanoparticle to the hydrogels had no effect on the springiness of the samples and in both complexes was 10 mm. The adhesion of the hydrogels showed similar behavior with hardness. The highest amount of the adhesion was related to κ-Carrageenan/Mg_0.99_Zn_0.01_O /NaCMC with −0.84 ± 0.17 (g. s). It reveals that the nanoparticle had a significant effect on the adhesion of the hydrogels. The addition of Mg_0.99_Zn_0.01_O particles led to a reduction in the pore size for κ-Carrageenan/Mg_0.99_Zn_0.01_O/NaCMC of the hydrogels. In the particle loaded hydrogels, the network of the gels moved close to each other. The smaller pore sizes in the structure of hydrogels imply a denser network, which was highly dependable with the equilibrium swelling ratio measurements. A denser network in the hydrogels reasonably presents their higher mechanical strength. These results showed that the presence of Mg_0.99_Zn_0.01_O particles network had a denser structure. Hence, a higher compressive strength achieved (*p* < 0.05 one-way ANOVA Tukey’s post hoc test). The covalent compositing of κ-Carrageenan/NaCMC hydrogels with Mg_0.99_Zn_0.01_O nanoparticles achieved higher mechanical strength rather than the nano particle-free hydrogel. The covalent compositing with particles increases the hardness and adhesion of the samples [45]. These findings were in line with the findings of a previous study where they examined the compressive properties of double-network hydrogels in grafting with silica nanoparticles. They reported that the elastic modulus of the hydrogels increased with the addition of nanoparticles [46].

### 3.6. TGA

**Figure 8:**
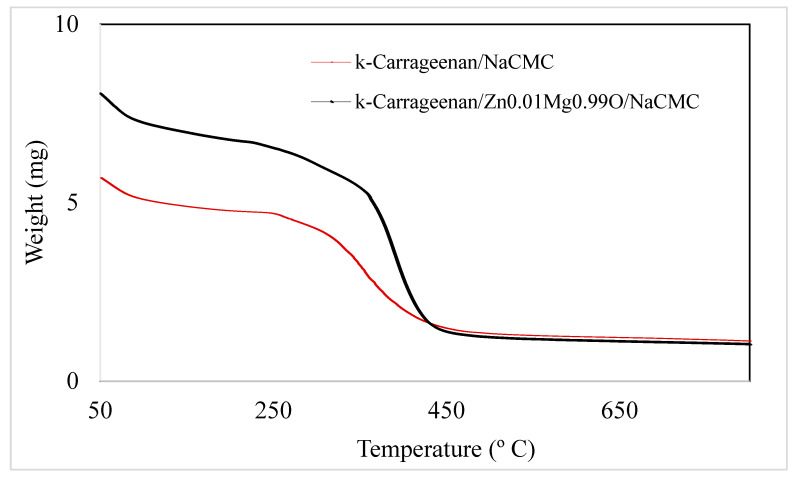
TGA curves of κ-Carrageenan/ Mg_0.99_Zn_0.01_O /NaCMC and of κ-Carrageenan/ NaCMC.

The TGA curves of the two carrageenan complexes are presented in Figure 8. The curves showed that approximately 80%–90% of the weight of the hydrogels was lost at 90° C. The second loss in the weight of the hydrogel was in the range of 250–390 °C for κ-Carrageenan /NaCMC and 250–420 °C for κ-Carrageenan/Mg_0.99_Zn_0.01_O/NaCMC. The first network degradation is related to the dehydration of the complex [46,47]. The entrapped water inside the matrix evaporates at this temperature. This water exists in an H-bonded form with the hydroxyl groups of glycosylic units along the polymer chain [37]. The increase in the decomposition temperature in κ-Carrageenan/Mg_0.99_Zn_0.01_O/NaCMC was related to the dispersion of the nanoparticle in the matrix. The second complex degradation was attributed to destroying the dense crosslinking of polymer chains inside the three-dimensional network [48]. The additional endothermic peak near 420 °C was characterized by the decomposition of Mg (OH)_2_ as an amorphous gradient to form MgO in the form of crystalline. The weight loss of the κ-Carrageenan/Mg_0.99_Zn_0.01_O/NaCMC sample was almost 17% [48].

### 3.7. Swelling Ratio

**Figure 9:**
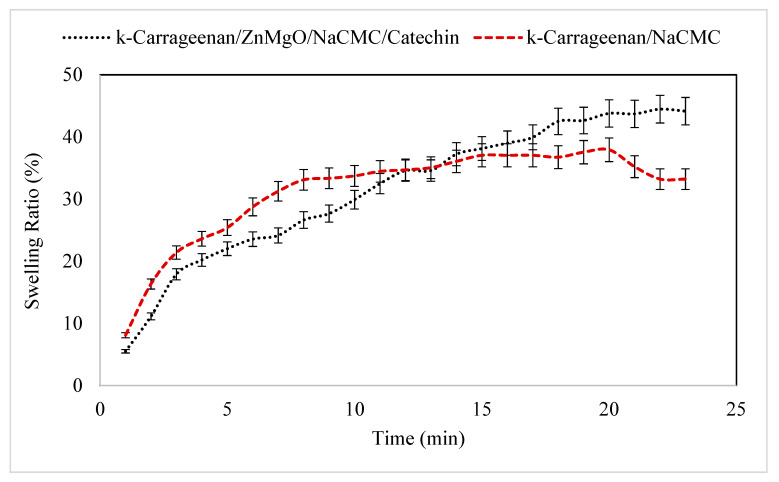
Swelling capacity of the two various composite (of κ-Carrageenan/Mg_0.99_Zn_0.01_O/NaCMC /Catechin and of κ-Carrageenan/NaCMC) hydrogel in distilled water.

Immersion of hydrogel in distilled water is aimed to increase the swelling in order to be loaded with different small-scale composites within its network such as drugs. The ion loading in nanocomposites depends on the swelling ability of hydrogels. Therefore, sodium salt of carboxymethylcellulose (CMC) was chosen to be mixed with κ-carrageenan to increase its swelling ability in distilled water. It is well known that NaCMC is the best-applied cellulose derivative [49]. It is made by treating cellulose with CH_2_ClCOOH, chloroacetic acid, and sodium hydroxide, which is in accordance with the Williamson etherification reaction. Among all cellulose derivatives, only the sodium salt (NaCMC), is a polyelectrolyte, which presents both ionic-strength and pH sensitivity variations. Furthermore, NaCMC has acceptable swelling capability [50,51]. The swelling rate of hydrogels is shown in Figure 9. Clearly, κ-Carrageenan/Mg_0.99_Zn_0.01_O/NaCMC/Catechin had the largest swelling ratio (44.15%) rather than the κ-Carrageenan/NaCMC (33.22%). The improvement in the swelling capacity of modified hydrogels can be due to more hydrophilic chains or hydration of functional groups on the polymeric chains [41,42]. During the experiment, it was clear that the hydrogels including Mg_0.99_Zn_0.01_O were physically harder gels (more stable) than the other hydrogels.

## 4. Conclusions

In this study, a novel hydrogel using Mg_0.99_Zn_0.01_O nanoparticles in the κ-Carrageenan/ NaCMC was prepared via green synthesis. The purpose of this synthesis was to provide a proper carrier for catechin loading in the hydrogel. Zeta potential analysis showed that functional ions transferring to the surface were affected by varying the Mg concentration value of the component. The XRD results confirmed that, based on the Scherror method, the size of κ-carrageenan/NaCMC/ Mg_0.99_Zn_0.01_O/Catechin was 18 nm and Mg_0.99_Zn_0.01_O was 20 nm, respectively. The addition of the Mg_0.99_Zn_0.01_O nanoparticle to the κ-Carrageenan/NaCMC hydrogel did not change the XRD pattern of the hydrogel remarkably, which indicates acceptable compatibility among κ-Carrageenan/NaCMC and the Mg_0.99_Zn_0.01_O nanoparticle. The surface morphology of the control hydrogel κ-Carrageenan/NaCMC was changed by loading the Mg_0.99_Zn_0.01_O nanoparticles. The FTIR analysis showed that Mg_0.99_Zn_0.01_O nanoparticles and catechin were grafted to carrageenan properly. TGA graphs of κ-Carrageenan/Mg_0.99_Zn_0.01_O/NaCMC and κ-Carrageenan/NaCMC hydrogels showed the distribution of nanoparticles to the network complex, which leads to increase the decomposition temperature in κ-Carrageenan/Mg_0.99_Zn_0.01_O/NaCMC. The compression test showed that, in the presence of Mg_0.99_Zn_0.01_O particles, the network had a denser structure. Hence, a higher compressive strength is achieved. The swelling ratio of κ-Carrageenan/ Mg_0.99_Zn_0.01_O/NaCMC/Catechin had the most swelling, which was attributed to the distributed nanoparticle in the matrix. According to the obtained results from different characterizations, the existence of catechin in the hydrogels had no effect on their structure. The purpose of loading catechin was to investigate the release studies that will be presented in future studies.

## Figures and Tables

**Table 1:** Compression test of the hydrogels.

Sample	Hardness (g)	Springiness (mm)	Adhesion (g. s)
κ-Carrageenan/NaCMC	76.61 ± 2.34	10	−0.73 ± 0.06
κ-Carrageenan/Mg_0.99_Zn_0.01_O/NaCMC	77.85 ± 3.40	10	−0.84 ± 0.17
